# Effect of Contact Pressure on the Performance of Carbon Nanotube Arrays Thermal Interface Material

**DOI:** 10.3390/nano8090732

**Published:** 2018-09-17

**Authors:** Yu Pei, Hongmei Zhong, Mengyu Wang, Peng Zhang, Yang Zhao

**Affiliations:** 1 Department of Precision Machinery & Precision Instrumentation, University of Science and Technology of China, Hefei 230022, China; peiyu@mail.ustc.edu.cn (Y.P.); fightingpsyche@163.com (H.Z.); wmy1083@mail.ustc.edu.cn (M.W.); zhp9036@mail.ustc.edu.cn (P.Z.); 2 CAS Key Laboratory of Mechanical Behavior and Design of Materials (LMBD), Department of Modern Mechanics, University of Science and Technology of China, Hefei 230026, China

**Keywords:** thermal interface material, contact pressure, vertically aligned carbon nanotube arrays, contact thermal conductance

## Abstract

Vertically aligned carbon nanotube (CNT) arrays are promising candidates for advanced thermal interface materials (TIMs) since they possess high mechanical compliance and high intrinsic thermal conductivity. Some of the previous works indicate that the CNT arrays in direct dry contact with the target surface possess low contact thermal conductance, which is the dominant thermal resistance. Using a phase sensitive transient thermo-reflectance (PSTTR) technique, we measure the thermal conductance between CNT arrays and copper (Cu) surfaces under different pressures. The experiments demonstrated that the contact force is one of the crucial factors for optimizing the thermal performance of CNT array-based TIMs. The experimental results suggest that the Cu-CNT arrays’ contact thermal conductance has a strong dependence on the surface deformation and has an order of magnitude rise as the contact pressure increases from 0.05 to 0.15 MPa. However, further increase of the contact pressure beyond 0.15 MPa has little effect on the contact thermal resistance. This work could provide guidelines to determine the minimum requirement of packaging pressure on CNT TIMs.

## 1. Introduction

With rapidly increasing power densities in electronic devices, thermal management is becoming a crucial issue in maintaining the reliability and performance. Inadequate power dissipation will raise the device temperature and shorten device lifetime. One way to improve the heat dispassion of devices is to find ultrahigh thermal conductivity materials for chips and heat sinks. Recently, Hu et al. synthesized semiconductor boron arsenide single crystals without detectable defects and with a thermal conductivity of 1300 W/mK at room temperature [1]. The graphite flakes-metal composites, reported by J. Narciso et al., possess a thermal conductivity between 294–390 W/mK, and are excellent materials for heat sinks [2]. However, with the further decrease of the thermal resistance of heat sinks, the thermal contact resistance between the chips and heat sinks due to the microscale surface roughness at the interface has become critical. A lot of effort has been put into searching for advanced thermal interface materials (TIMs) to enhance thermal conduction between chips and heat sinks. TIMs are used to replace the microscopic asperities between contact surfaces which contribute to the prominent thermal resistance [3]. Vertically aligned carbon nanotube (CNT) arrays are promising candidates for advanced TIMs since they possess high mechanical compliance [4,5,6,7,8] and high thermal conductivity at room temperature range [9,10,11,12,13].

Thermal resistance at the interface between dissimilar materials is composed of interface (boundary) thermal resistance due to mismatches in material properties and contact thermal resistance due to microscopic roughness at the interface. The contact at the carbon-metal interface in a pressure infiltrated carbon/metal composite is approximately ideal, and the interface thermal resistance at carbon-metal contact is predicted to be on the order of 10 MW/m^2^·K with acoustic mismatch model [14]. However, the actual contact area of the CNT array-metal interface is limited owing to the low volume fraction of the CNT array, which is a few percent in general. The roughness of the metal surface and the CNT top surface further limit the actual contact area. Therefore, contact thermal resistance is the main thermal resistance in this carbon-metal interface. Tong’s investigation showed that the thermal conductance at the direct contact glass-MWCNT interface was only about 0.09 MW/m^2^·K, and the contact between the CNTs to the mating surfaces was the dominant factor in the interface thermal resistance of CNT TIMs [15]. According to the study by Chu et al., both the surface roughness of CNT arrays and target surface affect the contact thermal resistance, and the contact conductance has an order of magnitude decrease as the target surface roughness increased to be comparable to the CNT array surface roughness [3]. Besides, Cola et al. developed a model to predict the thermal contact resistance of CNT array interfaces, which revealed that the thermal resistances at single-CNT contacts dominated the thermal transport across CNT array interfaces such that the effective thermal conductivity of the CNT array had little effect on interface resistance for moderate array heights (<30 μm) [16]. Therefore, improving the contact quality is crucial in achieving better thermal performance at interface with CNT TIMs. Researchers have shown that with assistance of either metallic bonding [15] or covalent bonding [17], the contact conductance at the CNT-surface interface could be improved significantly. Nevertheless, the contact formed by those bonding treatments at the interface was permanent, which might not be appropriate for some applications such as a thermal switch [18]. Furthermore, the huge pressure used in the bonding process makes CNT arrays completely densified and might lose the mechanical compliance.

Generally, packaging pressure plays an important role in the performance of TIMs, especially for CNT arrays. Due to the mechanical compliance of the CNT array, the contact area can be improved by applying enough pressure, and thus enhancing the thermal transport at interface. On one hand, low pressure means poor contact and high thermal resistance, on another hand, excessive pressure may cause irreparable damage to chips and CNT arrays. So there should be an optimal contact force for CNT TIMs and the packaging pressure needs to be precisely controlled within the optimal pressure range. However, investigation into the influence of contact pressure on interface thermal resistance regarding CNT TIMs is limited. Much effort has been made to study mechanical behavior of vertically aligned CNT arrays [19], but those works primarily focused on the vertical buckling rather than the deformation of the upper surface of the CNTs. In this work, we systematically studied the effect of contact pressure on the thermal performance of CNT arrays in dry contact with copper surfaces. Using a phase sensitive transient thermo-reflectance (PSTTR) technique, we were able to distinguish the thermal conductance at Cu/CNT arrays interface from the thermal properties of CNT array layer itself. Here we obtained the relationship between contact thermal conductance and contact pressure, which indicates that the increase of contact pressure could improve the thermal performance of CNT arrays TIMs. And an optimum contact pressure was obtained since the contact thermal conductance tends to saturate after the pressure is greater than 0.15 MPa. With the advantage of avoiding bonding processing, the results could benefit the practical integration of CNT arrays for thermal management in microelectronic devices.

## 2. Materials and Methods

### 2.1. CNT Arrays Synthesis and Characterization

**Figure 1:**
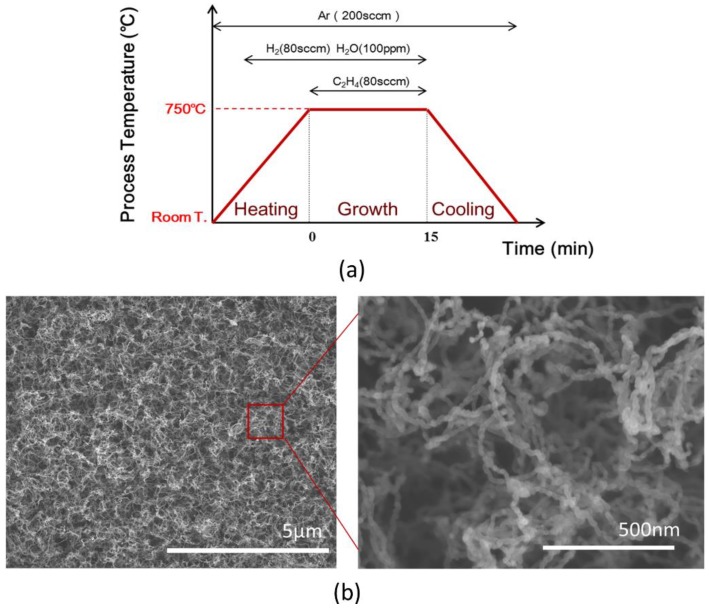
(**a**) The sketch of the chemical vapor deposition (CVD) process; (**b**) Scanning electron microscopy (SEM) images of carbon nanotube (CNT) array top surface.

Vertically aligned CNT arrays on silicon (Si) wafer (CSTC, Suzhou, China, P-type) were prepared by the water-assisted chemical vapor deposition (CVD) method with ferrum (Fe) as catalyst as shown in Figure 1a [20]. A 20-nm buffer layer of aluminium oxide (Al_2_O_3_) and a 1-nm layer of Fe were subsequently deposited onto the Si substrate by sputtering (Kurt J. Lesker. LAB 18, Jefferson Hills, PA, USA) and thermal evaporation (VNANO, Beijing, China, VZZ-300). The CVD process was done under an argon (Ar) and hydrogen (H_2_) environment with ethylene (C_2_H_4_) as feedstock in a tube furnace (KEJING, Hefei, China, OTF-1200X). A mixture of Ar (120 sccm), H_2_ (80 sccm), and a small amount of water vapor (~100 ppm) was supplied into the reaction tube while the tube was heated to 750 °C. Once the furnace reached the target temperature, C_2_H_4_ gas with a flow rate of 80 sccm was introduced into the gas stream to start the growth process. The growth time was controlled to be 10 to 15 min. Then the supply of C_2_H_4_, H_2_ and water vapor was terminated and the sample was cooled down to room temperature in an Ar environment. By changing the time of the flow of ethylene, CNT arrays with lengths of 200 and 500 μm were obtained, respectively. It has been shown that both the contact conductance and the effective thermal conductivity have direct relationship with volume fraction of CNT arrays [21]. In this work, the array volume fraction of the CNT arrays was controlled to be around 1.8% which was determined by the ratio of the array mass density to the individual CNT mass density [22], 2.1 g/cm^3^. Scanning electron microscopy (SEM) (Hatachi, Tokyo, Japan, SU8220) image (Figure 1b) at 3 KV voltage shows the morphology of the upper surface of CNT arrays. The diameter of individual carbon nanotubes are 10 to 20 nanometers. The mechanical behaviors of the prepared CNT arrays were studied with an electronic compression testing machine (REGER, Shenzhen, China, RGM-6005T). The compression head was set to retreat at a speed of 5 mm/min. And an atomic force microscopy (AFM) (Bruker, Billerica, MA, USA, Demension Icon) was used to characterize the roughness of the upper surface of the CNT both before and after the pressing.

### 2.2. Thermal Measurement Setup

**Figure 2:**
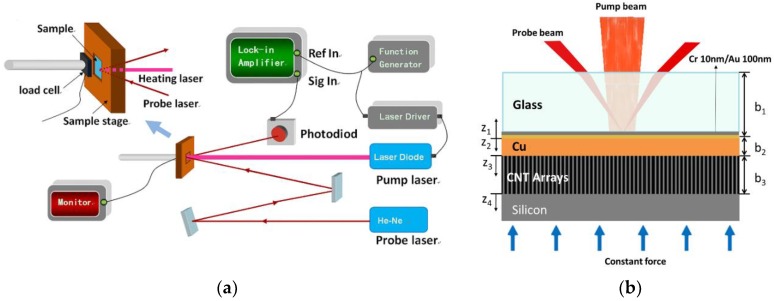
(**a**) Experimental setup of phase sensitive transient thermo-reflectance measurement for thermal performances; (**b**) Sample structure with pump and probe beam path.

The heat transfer properties of Cu/CNT interface were measured using phase sensitive transient thermo-reflectance (PSTTR) method [15,23]. Similar to time-domain thermo-reflectance (TDTR), [24,25] PSTTR is an optical method based on the thermal reflectance effect. Instead of using a femto-second laser as heating source, it uses a modulated continuous wave laser to provide heat flux at sample surfaces. The modulation frequency is kept at the kHz range to ensure the penetration of the heat wave through the several microns thick copper layer and few hundred microns thick CNT array in sequence. The experimental apparatus is illustrated in Figure 2a.

The sample was heated by a diode laser (RPMC, O’Fallen, MO, USA, LDX-3315-808 with nominal wavelength of 808 nm and maximum output power ~1 W) with intensity sinusoidally modulated at frequency ω. Taking into account the factors of thermal penetration depth and signal to noise ratio, the modulation frequency ω was set within 3000–6000 Hz range and controlled by a function generator. Figure 2b shows the sample configuration for thermal characterization. A piece of glass was sequentially deposited with 10 nm chromium (Cr) as adhesion promoter and 100 nm gold (Au) for its high thermo-reflectance coefficient at the probe laser wavelength (632.8 nm). Then a five-micron thickness of copper was deposited onto the Au side of the glass wafer by thermal evaporation. The CNT array on the silicon substrate was adhered to the Cu side of the glass wafer with a controlled pressure by a load cell.

### 2.3. Measurement Principle

The heating laser beam passed through the glass plate and was focused onto the Cr/Au layer with beam size around 1 mm × 2 mm as shown in Figure 2b. The oscillating heat flux propagated through the sample causing temperature oscillation at the Cr/Au surface at same frequency. Due to the thermo-reflectance effect, the reflectivity of Au surface oscillates accordingly. A He-Ne laser (CNI Laser, Changchun, China, 633 nm, 5 mW) beam with constant intensity was concentrically focused on to the Cr/Au layer with beam size around 100 μm in radius as a probe beam. The intensity of reflected probe beam was therefore modulated by the temperature at Au surface. A photo detector captured the reflected probe beam, and the signal was sent to a lock-in amplifier (Stanford Research Systems, Sunnyvale, CA, USA, SR850) to extract the intensity oscillation at frequency ω. The thermal impedance of the sample caused a phase lag of the temperature oscillation compared with the heating signal. Therefore, by analyzing the phase lags according to modulation frequencies, thermal properties of the sample could be determined.

The conduction of heat within the samples can be simplified as one-dimensional heat transfer, since the heating area on the Cr/Au surface was much larger than the probe beam size and penetration depth of the heat wave. Therefore, a three layer 1-D transient heat conduction model was constructed to obtain the thermal properties according to the sample structure in Figure 2b. The governing equation [26] and their boundary conditions are

(1) $$\frac{1}{\alpha_{j}}\frac{\partial T_{j}\left(z,t\right)}{\partial t}=\frac{\partial^{2}T_{j}\left(z,t\right)}{\partial z_{j}{}^{2}}$$

(2) $$-k_{1}{\frac{\partial T_{1}}{\partial Z_{1}}|}_{z_{1}=0}+h_{glass–Cu}\left[T_{1}\left(0\right)-T_{2}\left(0\right)\right]=Qe^{-i\omega t}$$

(3) $$-k_{2}{\frac{\partial T_{2}}{\partial Z_{2}}|}_{z_{2}=0}=h_{glass–Cu}\left[T_{1}\left(0\right)-T_{2}\left(0\right)\right]$$

(4) $$-k_{2}{\frac{\partial T_{2}}{\partial Z_{2}}|}_{z_{2}=b_{2}}=h_{Cu-CNTs}\left[T_{2}\left(b_{2}\right)-T_{3}\left(0\right)\right]=-k_{3}{\frac{\partial T_{3}}{\partial Z_{3}}|}_{z_{3}=0}$$

(5) $$-k_{3}{\frac{\partial T_{3}}{\partial Z_{3}}|}_{z_{3}=b_{3}}=h_{CNTs–Si}\left[T_{3}\left(b_{3}\right)-T_{4}\left(0\right)\right]=-k_{4}{\frac{\partial T_{4}}{\partial Z_{4}}|}_{z_{4}=0}$$

(6) $$-k_{1}{\frac{\partial T_{1}}{\partial Z_{1}}|}_{z_{1}=b_{1}}=0,\;-k_{4}{\frac{\partial T_{4}}{\partial Z_{4}}|}_{z_{4}=b_{4}}=0$$

where:

*j* represents the *j*^th^ layer (1 = glass, 2 = copper, 3 = CNTs, 4 = silicon),
*α* is the thermal diffusivity,
*T* is the temperature,
*k* is cross-plane effective thermal conductivity,
*ω* is the frequency of pump laser,
*h* is the interface thermal conductance.

## 3. Results and Discussion

**Figure 3:**
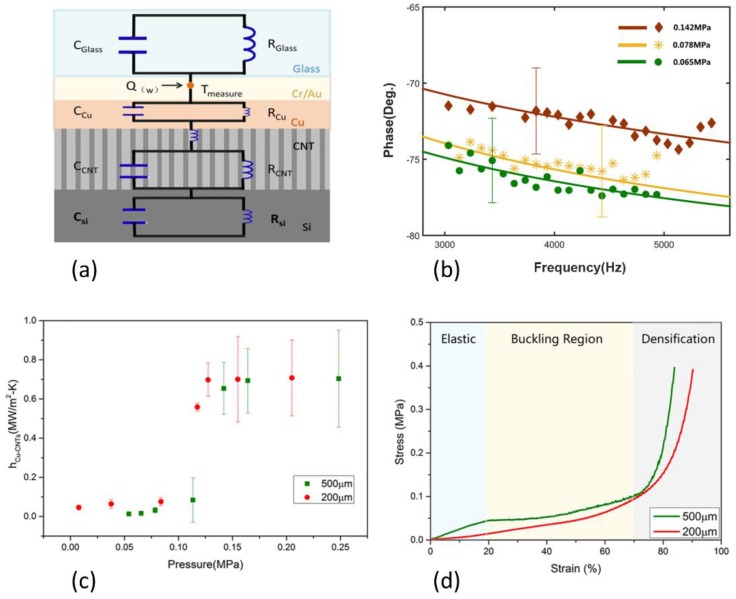
(**a**) Simplified heat transfer mechanism within the sample illustrated in thermal circuit; (**b**) Experimentally measured phase responses (various points) of various contact force along with their best-fit solutions (solid lines) versus excitation frequency; (**c**) Cu-CNT arrays contact thermal conductance versus contact pressure; (**d**) Experimental uniaxial stress–strain responses of vertically aligned CNT Arrays.

The thermal circuit of the system is shown in Figure 3a. The heat flux from the laser can be divided into two components, a steady state part and an oscillatory or AC part given as *Qe^−iωt^*. The phase lag of the temperature oscillation at the Au/Cr-coated surface of the glass is analytically solved as a function of the driving frequency to fit with the measured phase response. The contact conductance between Cr/Au coated glass and the Cu film is set to be 10^9^ W/m^2^·K according to the measured metal/metal thermal contact conductance [27]. The penetration depth of a thermal wave in CNT is about a few hundred microns at our measurement frequency domain. The contact conductance between CNT arrays and the silicon substance is set to be 10^6^ W/m^2^·K according to previous reports [15]. The volumetric heat capacity of the CNT arrays, C_CNTs_, is equal to the mass density times the heat capacity of the individual MWCNT, 700 J/kg·K [28].

The measured phase responses (various points) of the samples with 500 μm high CNT arrays versus excitation frequency are shown in Figure 3b. Five hundred phase data are obtained for each frequency point, and the error bar is the standard deviation of these data. The best-fit solutions are calculated by MATLAB models. The experimental results are then fitted to theoretical model (solid lines in Figure 3b) using a best fit scenario to extract the contact thermal conductance at Cu/CNT arrays interface (*h_Cu-CNTs_*) and the effective thermal conductivity of CNT arrays (*k_CNTs_*). The error bars of *h_Cu-CNTs_* and *k_CNTs_* came from the fitting of the experimental data varying according to the standard deviation of the measurements (the error bar in Figure 3b). The fitted value of *k_CNTs_* is around 18 ± 6 W/m·K. Considering an array volume fraction of 1.8% of the MWCNTs, the effective thermal conductivity of the MWCNT array qualitatively matches with the previous measurement of an individual MWCNT, 650–3000 W/mK [13,29]. The thermal conductance at interface for various contact force are listed in Table 1, and plotted in Figure 3c which shows three distinct stages along the loading process. In the initial stage at pressure below 0.1 MPa, *h_Cu-CNTs_* increases slowly with the contact force. Then as contact pressure keeps increasing, there is a sharp rise in *h_Cu-CNTs_*. In the final plateau, contact pressure has almost no influence on *h_Cu-CNTs_*.

**Figure 4:**
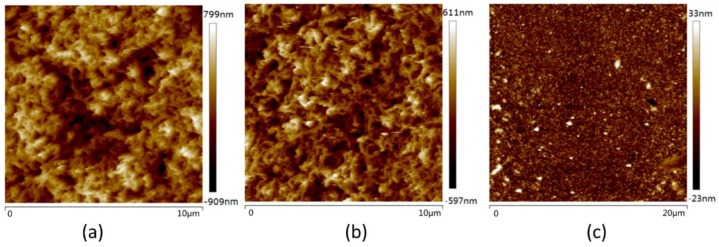
(**a**) The atomic force microscopy (AFM) topography profile of upper surface of CNT arrays before loading. The Ra roughness is 197 nm; (**b**) The AFM topography profile of upper surface of CNT arrays after loading. The Ra roughness is 136 nm; (**c**) The AFM topography profile of copper. The Ra roughness is 5.67 nm.

**Figure 5:**
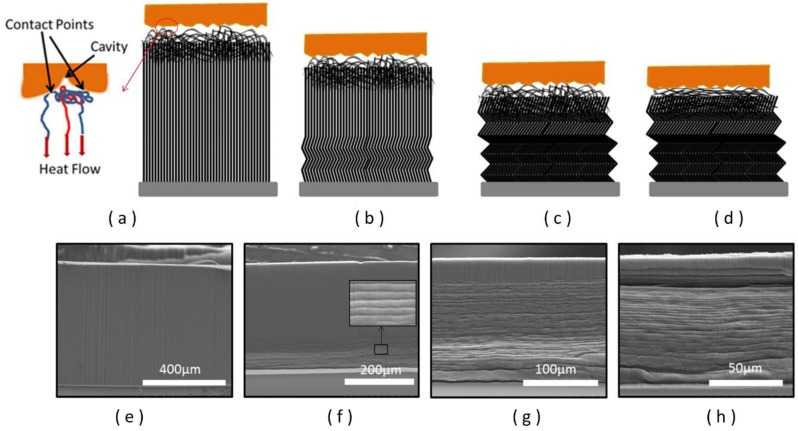
(**a**–**d**) Schematic diagrams showing the cross section of CNT array in contact with a copper surface under different compression; (**e**–**h**) SEM images of CNT arrays after 0, 0.06, 0.12, 0.5MPa pressure compression, respectively.

The fact that the thermal contact conductance has a strong dependence on contact force reveals that the morphology and the roughness at the contact surfaces impair the effective contact between CNT arrays and target surfaces significantly. The top surface of CNT array is examined under an AFM scanner before and after compression testing with 0.5 MPa pressure, and a decrease of the arithmetical mean roughness (Ra) from 197 to 136 nm is observed (Figure 4a,b). If considering that the deformation of the top of the CNT is not completely plastically deformed, the actual roughness will be lower in the loaded state. The Ra roughness of the evaporated Cu top surface is 5.67 nm (Figure 4c). Figure 1b shows that on the top surface of CNT arrays, the nanotubes form an entangling surface layer like a canopy as a result of van der Waals interactions. As depicted in Figure 5a, a CNT that is directly facing a cavity (shown in red) cannot make contact due to restriction force from adjacent tubes (shown in blue). And those tubes that are not in direct contact to the mating surfaces cannot participate in heat conduction. Hence, the CNT/Cu interface shows a poor thermal contact at the beginning stage.

The slowly increased thermal contact at the initial stage distinguishes CNT/Cu interfaces from general dry contact surfaces as they usually experience immediate increase with contact force. The behavior is due to the unique mechanical response of CNT arrays under pressure. Figure 3d shows the stress (the applied force divided by the array’s area) versus strain (the compressed distance relative to the array’s thickness) curves for the CNT arrays under compression. The compression process is generally including an initial elastic region, a buckling region marked by a plateau and a final densification, which is consistent with previous research [19]. SEM images (Figure 5e–h) illustrate that CNT arrays form sequential buckling-like folds, which emanates from the bottom and propagates toward the top. The average buckling wavelength is around 2 μm. Previous study has revealed that the sequence of bottom-to-top buckling was driven by the relative local density [30]. As can be evaluated from the level of the stresses, the slowly increasing stage shown in Figure 3c corresponds to the buckling region in Figure 3d. As can be seen, the pressure mainly contributes to the buckling rather than the deformation of the top surface of CNT arrays at this stage as illustrated in Figure 5a,b and therefore, does not improve the thermal contact at interface significantly.

The buckling process ends roughly at 0.1 MPa stress level (Figure 3d), and a sharp increase of the thermal conductance at CNT/Cu interface is observed simultaneously (Figure 3c), since the top surface of the CNT array starts to experience large deformation and comply with the roughness at interface (Figure 5c,d). CNT arrays with heights of 200 μm and 500 μm have shown a similar surging trend. However, for 500 μm CNT array, the pressure needed to complete the buckling process would be larger than 200 μm one, as shown in the stress–strain curve. In other words, longer CNT arrays require a higher pressure to enter the stage of rapid rise. In the final plateau, the value of *h_Cu-CNTs_* tends to be stable owing to the fact that all the deformations have been completed in this phase. Therefore, the pressure at the inflection point of the second and the third stage may be considered to be an optimum packaging pressure.

## 4. Conclusions

In this work, we investigated the effect of contact pressure on the performance of carbon nanotube arrays thermal interface material. CNT arrays with two different heights were both measured for contact thermal conductance by phase sensitive transient thermo-reflectance method under different loads. The entanglement of the upper surface of the CNT array weakens its contact with the target surface, which can be notably alleviated by raising the contact pressure. In addition, the experimental results indicate that the buckling of the CNT arrays defers the improvement of thermal contact at interface with contact pressure, and the deformation of the upper surface of CNT arrays mainly occurred at the end of the buckling process. As the contact force increases from 0.1 to 0.15 MPa, *h_Cu-CNTs_* enhances sharply. Worth mentioning, however, is that *h_Cu-CNTs_* tends to level off after 0.15 MPa, which should be the optimal pressure applied during the packaging of the electronic components for the studied CNT arrays. However, it should be noted that the height and density of the CNT arrays could affect their mechanical response under compression significantly, and thus the optimal packaging pressure is sensitive to the arrays that are in use. Moreover, the roughness of target surface is also an important factor at thermal interface, thus the effect of contact pressure of a thermal interface with various roughness should also be studied carefully.

## Figures and Tables

**Table 1:** Cu-CNT arrays contact thermal conductance versus contact pressure.

Height of CNT Array	Parameters	Values						
500 μm	Pressure	0.054	0.065	0.078	0.113	0.142	0.164	0.248
	(MPa)							
	*h_Cu-CNTs_*	0.007	0.008	0.024	0.084	0.654	0.694	0.704
	(MW/m^2^·K)							
200 μm	Pressure	0.008	0.038	0.084	0.118	0.128	0.155	0.205
	(MPa)							
	*h_Cu-CNTs_*	0.046	0.064	0.076	0.559	0.698	0.700	0.708
	(MW/m^2^·K)							

## References

[1] Kang J.S., Li M., Wu H., Nguyen H., Hu Y. (2018). Experimental observation of high thermal conductivity in boron arsenide. Science.

[2] Prieto R., Molina J.M., Narciso J., Louis E. (2011). Thermal conductivity of graphite flakes-SiC particles/metal composites. Compos. Part. A-Appl. Sci. Manuf..

[3] Chu R.-S., Zhao Y., Grigoropoulos C.P. (2012). Effect of copper surface roughness on thermal conductance of copper/carbon nanotube array interface. 13th InterSociety Conference on Thermal and Thermomechanical Phenomena in Electronic Systems, Proceedings of the 13th InterSociety Conference on Thermal and Thermomechanical Phenomena in Electronic Systems, San Diego, CA, USA, 30 May–1 June 2012.

[4] Cao A. (2005). Super-compressible foamlike carbon nanotube films. Science.

[5] Zhao Y., Tong T., Delzeit L., Kashani A., Meyyappan M., Majumdar A. (2006). Interfacial energy and strength of multiwalled-carbon-nanotube-based dry adhesive. J. Vac. Sci. Technol. B Nanotechnol. Microelectron. Mater. Process. Meas. Phenom..

[6] Suhr J., Victor P., Ci L., Sreekala S., Zhang X., Nalamasu O., Ajayan P.M. (2007). Fatigue resistance of aligned carbon nanotube arrays under cyclic compression. Nat. Nanotechnol..

[7] Tong T., Zhao Y., Delzeit L., Kashani A., Meyyappan M., Majumdar A. (2008). Height independent compressive modulus of vertically aligned carbon nanotube arrays. Nano Lett..

[8] Qu L., Dai L., Stone M., Xia Z., Wang Z.L. (2008). Carbon nanotube arrays with strong shear binding-on and easy normal lifting-off. Science.

[9] Berber S., Kwon Y.-K., Tománek D. (2000). Unusually high thermal conductivity of carbon nanotubes. Phys. Rev. Lett..

[10] Fujii M., Zhang X., Xie H., Ago H., Takahashi K., Ikuta T., Abe H., Shimizu T. (2005). Measuring the thermal conductivity of a single carbon nanotube. Phys. Rev. Lett..

[11] Pop E., Mann D., Wang Q., Goodson K., Dai H. (2006). Thermal conductance of an individual single-wall carbon nanotube above room temperature. Nano Lett..

[12] Pettes M.T., Shi L. (2009). Thermal and structural characterizations of individual single-, double-, and multi-walled carbon nanotubes. Adv. Funct. Mater..

[13] Kim P., Shi L., Majumdar A., McEuen P.L. (2001). Thermal transport measurements of individual multiwalled nanotubes. Phys. Rev. Lett..

[14] Caccia M., Rodríguez A., Narciso J. (2014). Diamond surface modification to enhance interfacial thermal conductivity in Al/diamond composites. JOM.

[15] Tong T., Zhao Y., Delzeit L., Kashani A., Meyyappan M., Majumdar A. (2007). Dense vertically aligned multiwalled carbon nanotube arrays as thermal interface materials. IEEE Trans. Compon. Packag. Technol..

[16] Cola B.A., Xu J., Fisher T.S. (2009). Contact mechanics and thermal conductance of carbon nanotube array interfaces. Int. J. Heat Mass Transf..

[17] Kaur S., Raravikar N., Helms B.A., Prasher R., Ogletree D.F. (2014). Enhanced thermal transport at covalently functionalized carbon nanotube array interfaces. Nat. Commun..

[18] Cho J., Richards C., Bahr D., Jiao J., Richards R. (2008). Evaluation of contacts for a MEMS thermal switch. J. Micromech. Microeng..

[19] Pathak S., Paris O. (2016). Collective behaviour of vertically aligned carbon nanotubes: from a single tube towards complex networks. Structure and Multiscale Mechanics of Carbon Nanomaterials.

[20] Zhao Y., Chu R.-S., Grigoropoulos C.P., Dubon O.D., Majumdar A. (2016). Array volume fraction-dependent thermal transport properties of vertically aligned carbon nanotube arrays. J. Heat Transf..

[21] Ohsone Y., Wu G., Dryden J., Zok F., Majumdar A. (1999). Optical measurement of thermal contact conductance between wafer-like thin solid samples. J. Heat Transf..

[22] Lu Q., Keskar G., Ciocan R., Rao R., Mathur R.B., Rao A.M., Larcom L.L. (2006). Determination of carbon nanotube density by gradient sedimentation. J. Phys. Chem. B.

[23] Feng X., King C., Narumanchi S. (2016). General multilayer heat transfer model for optical-based thermal characterization techniques. Int. J. Heat Mass Transf..

[24] Jiang P., Huang B., Koh Y.K. (2016). Accurate measurements of cross-plane thermal conductivity of thin films by dual-frequency time-domain thermoreflectance (TDTR). Rev. Sci. Instrum..

[25] Cahill D.G. (2004). Analysis of heat flow in layered structures for time-domain thermoreflectance. Rev. Sci. Instrum..

[26] Incropera F., Dewitt D. (1985). Introduction to heat transfer.

[27] Gundrum B.C., Cahill D.G., Averback R.S. (2005). Thermal conductance of metal-metal interfaces. Phys. Rev. B.

[28] Mizel A., Benedict L.X., Cohen M.L., Louie S.G., Zettl A., Budraa N.K., Beyermann W.P. (1999). Analysis of the low-temperature specific heat of multiwalled carbon nanotubes and carbon nanotube ropes. Phys. Rev. B.

[29] Choi T.Y., Poulikakos D., Tharian J., Sennhauser U. (2005). Measurement of thermal conductivity of individual multiwalled carbon nanotubes by the 3-ω method. Appl. Phys. Lett..

[30] Pathak S., Mohan N., Decolvenaere E., Needleman A., Bedewy M., Hart A.J., Greer J.R. (2013). Local relative density modulates failure and strength in vertically aligned carbon nanotubes. ACS Nano.

